# Assessment of antibiotic resistance in *Klebsiella pneumoniae* exposed to sequential in vitro antibiotic treatments

**DOI:** 10.1186/s12941-016-0173-x

**Published:** 2016-12-09

**Authors:** Jeongjin Kim, Ara Jo, Ekachai Chukeatirote, Juhee Ahn

**Affiliations:** 1 Department of Medical Biomaterials Engineering, Kangwon National University, Chuncheon, Gangwon 24341 South Korea; 2 School of Science, Mae Fah Luang University, Chiang Rai, 57100 Thailand; 3 Institute of Bioscience and Biotechnology, Kangwon National University, Chuncheon, Gangwon 24341 South Korea

**Keywords:** β-Lactamase, Efflux pump system, Meropenem, Ciprofloxacin, Antibiotic resistance

## Abstract

**Background:**

Bacteria treated with different classes of antibiotics exhibit changes in susceptibility to successive antibiotic treatments. This study was designed to evaluate the influence of sequential antibiotic treatments on the development of antibiotic resistance in *Klebsiella pneumoniae* associated with β-lactamase and efflux pump activities.

**Methods:**

The antibiotic susceptibility, β-lactamase activity, and efflux activity were determined in *K*. *pneumoniae* grown at 37 °C by adding initial (0 h) and second antibiotics (8 or 12 h). Treatments include control (CON; no first and second antibiotic addition), no initial antibiotic addition followed by 1 MIC ciprofloxacin addition (CON-CIP), no initial antibiotic addition followed by 1 MIC meropenem addition (CON-MER), initial 1/4 MIC ciprofloxacin addition followed by no antibiotic addition (1/4CIP-CON), initial 1/4 MIC ciprofloxacin addition followed by 1 MIC ciprofloxacin addition (1/4CIP-CIP), and initial 1/4 MIC ciprofloxacin addition followed by 1 MIC meropenem addition (1/4CIP-MER).

**Results:**

Compared to the CON, the initial addition of 1/4 MIC ciprofloxacin inhibited the growth of *K*. *pneumoniae* throughout the incubation period. The ciprofloxacin treatments (CON-CIP and 1/4CIP-CIP) showed significant reduction in the number of *K*. *pneumoniae* cells compared to meropenem (CON-MER and 1/4CIP-MER). The 1/4CIP-CIP achieved a further 1 log reduction of *K*. *pneumoniae*, when compared to the 1/4CIP-CON and 1/CIP-MER. The increase in sensitivity of *K*. *pneumoniae* to cefotaxime, kanamycin, levofloxacin, nalidixic acid was observed for CON-CIP. Noticeable cross-resistance pattern was observed at the 1/4CIP-CIP, showing the increased resistance of *K*. *pneumoniae* to chloramphenicol, ciprofloxacin, kanamycin, levofloxacin, nalidixic acid norfloxacin, sulphamethoxazole/trimethoprim, and tetracycline. The levels of β-lactamase activities were estimated to be 8.4 μmol/min/ml for CON, 7.7 μmol/min/ml for 1/4CIP-CON and as low as 2.9 μmol/min/ml for CON-CIP. Compared to the absence of phenylalanine-arginine-β-naphthylamide (PAβN), the fluorescence intensity of EtBr was increased in *K*. *pneumoniae* cells treated at the CON, CON-CIP, and CON-MER in the presence of PAβN. However, the efflux pump activity remained in *K*. *pneumoniae* cells treated at the 1/CIP, 1/CIP–CIP, and 1/CIP-MER in the presence of PAβN.

**Conclusion:**

The results suggest that the pre-exposed antibiotic history, treatment order, and concentrations influenced the development of multiple antibiotic resistant associated with β-lactamase and efflux pump activities. This study highlights the importance of antibiotic treatment conditions, which would be taken into consideration when new antibiotic strategy is designed to prevent antibiotic resistance.

## Background

The overuse, underuse, and misuse of antibiotics have become major causes of the development of antibiotic resistance in bacteria [1]. The emergence and spread of antibiotic-resistant bacteria has been a growing concern over the last decade, which can lead to serious clinical and public health problems. *Klebsiella pneumoniae*, an opportunistic pathogen, is mainly responsible for nosocomial infections with high morbidity and mortality [2]. Recently, the rapid emergence of extended-spectrum β-lactamase (ESBL) producing *K*. *pneumoniae* has significantly increased the risk of developing serious nosocomial and community-acquired infections worldwide [3, 4]. Furthermore, multidrug-resistant *K*. *pneumoniae* strains can cause treatment failure with current antibiotic therapy [2].

The proposed resistance mechanisms of *K*. *pneumoniae* against different classes of antibiotics include release of antibiotic-inactivating enzymes, modification of antibiotic target sites, change in membrane permeability, activation of efflux pump systems, and alteration of metabolic pathways [2, 5]. Among these mechanisms, the enzymatic degradation and efflux pump systems play an important role in the development of multidrug resistance in *K*. *pneumoniae* [6]. *K*. *pneumoniae* strains produce enzymes, including extended-spectrum β-lactamases, metallo-β-lactamases, oxacillinases, and carbapenemases, that can degrade β-lactam antibiotics. The efflux pumps, belonging to the resistance-nodulation-division (RND) family, can extrude amphiphilic and charged antibiotics such as β-lactams, fluoroquinolones, and aminoglycosides [2].

The sequential and combination antibiotic therapies have currently been used to reduce not only the evolution of multidrug resistant bacteria but also the levels of antibiotics used in the treatment of bacterial infection [7–9]. The decreased selection pressure occurs when antibiotics are treated in appropriate order, which can prevent the emergence and spread of multidrug resistance [10]. From the practical viewpoint of antibiotic effectiveness, however, there is an important challenging question of whether the treatment history can cause potential carry-over effects on the additional antibiotic therapy. The pre-exposure to antibiotics influences the acquisition of resistance to second-line antibiotics [11]. The beneficial and adverse effects of current therapeutic approaches for treating bacterial infections are still under debate. Therefore, the objective of this study was to evaluate the impact of sequential antibiotic treatments on the antibiotic susceptibility and resistance mechanisms of *K*. *pneumoniae* in association with β-lactamase and efflux pump activities.

## Methods

### Bacterial strain and culture condition

Strain of *K*. *pneumoniae* ATCC 23357 was purchased from American Type Culture Collection (ATCC, Manassas, VA, USA). The strain was cultured in trypticase soy broth (TSB; BD, Becton, Dickinson and Co., Sparks, MD, USA) at 37 °C for 18 h. After cultivation, the culture was centrifuged at 3000×*g* for 20 min at 4 °C. The harvested cells were washed twice with phosphate-buffered saline (PBS, pH 7.2) and diluted to 10^8^ CFU/ml for assays.

### Antibiotic susceptibility assay

The susceptibility of *K*. *pneumoniae* to ciprofloxacin and meropenem was evaluated according to the Clinical Laboratory Standards Institute (CLSI) procedure with slight modification [12]. All antibiotics used in this study were purchased from Sigma Chemicals (St. Louis, MO, USA). Antibiotic stock solutions were prepared by dissolving in glacial acetic acid for ciprofloxacin and distilled water for meropenem to obtain a final concentration of 10.24 mg/ml. The stock solutions of ciprofloxacin and meropenem (100 μl each) were serially (1:2) diluted to concentrations ranging from 2 to 0 μg/ml with TSB in 96-well flat-bottomed polystyrene microtiter plates (BD Falcon, San Jose, CA, USA). The test strain was inoculated at 10^5^ CFU/ml and incubated at 37 °C for 18 h. Minimum inhibitory concentrations (MICs) of ciprofloxacin and meropenem were defined as the lowest concentration of each antibiotic at which no visible cell growth was observed.

### Dynamic time-kill curve analysis

Time-kill curve assay was carried out to determine the antibiotic activities of ciprofloxacin and meropenem against *K*. *pneumoniae* using a sequential treatment scheme as described in Table 1. The initial population (10^5^ CFU/ml) of *K*. *pneumoniae* was inoculated at 37 °C in TSB treated with (Treatments 1, 2, and 3) and without antibiotic (Treatments 4, 5, and 6). After being reached an optical density (OD) of 0.5, the non-adapted and ciprofloxacin-adapted *K*. *pneumoniae* cells were further treated with no (Treatments 1 and 4) and the increased concentrations of ciprofloxacin (Treatments 2 and 5) and meropenem (Treatments 3 and 6). The survival curves were measured at 600 nm at every 4 h interval throughout the incubation period. The cultured cells were collected for further analyses, including bacterial enumeration, disk diffusion, lactamase activity, and cartwheel tests.

**Table 1:** Sequential antibiotic treatments used in this study

Treatment	Antibiotic addition (h)		Abbreviation	Description
	0 h	8 or 12 h		
1	No (0)	No (8)	CON	No first and second antibiotic addition
2	No (0)	1 MIC CIP (8)	CON-CIP	No initial antibiotic addition followed by 1 MIC ciprofloxacin addition
3	No (0)	1 MIC MER (8)	CON-MER	No initial antibiotic addition followed by 1 MIC meropenem addition
4	1/4 MIC CIP (0)	No (12)	1/4CIP-CON	Initial 1/4 MIC ciprofloxacin followed by no antibiotic addition
5	1/4 MIC CIP (0)	1 MIC CIP (12)	1/4CIP-CIP	Initial 1/4 MIC ciprofloxacin followed by 1 MIC ciprofloxacin addition
6	1/4 MIC CIP (0)	1 MIC MER (12)	1/4CIP-MER	Initial 1/4 MIC ciprofloxacin followed by 1 MIC meropenem addition

CON, CIP, and MER denote no antibiotic addition, ciprofloxacin, and meropenem, respectively

### Microbial analysis

The collected cells were serially diluted (1:10) with PBS and then plated on trypticase soy agar (TSA) using an Autoplate Spiral Plating System (Spiral Biotech Inc., Norwood, MA, USA). The plates were incubated at 37 °C for 24–48 h to enumerate viable cells using a QCount Colony Counter (Spiral Biotech Inc.). Log reduction (*N*_TRT_/*N*_CON_) was calculated for each treatment as compared to the control (CON). *N*_CON_ and *N*_TRT_ represent the numbers of control and treatments after incubation, respectively.

### Disk-diffusion assay

The antibiotic susceptibility of cultured *K*. *pneumoniae* was determined by the disk-diffusion test. The cultured cells were evenly spread-plated on TSA and then allowed to dry for 5 min. Antibiotic disks (Becton, Dickinson and Company, NJ, USA), including cefotaxime (CTX; 30 μg), chloramphenicol (CHL; 30 μg), ciprofloxacin (CIP; 5 μg), kanamycin (KAN; 30 μg), levofloxacin (LEV; 5 μg), nalidixic acid (NAL; 30 μg), norfloxacin (NOR; 10 μg), sulphamethoxazole/trimethoprim (S/T; 25 μg), and tetracycline (TET; 30 μg), were placed on the surface of the agar using sterilized forceps. After incubation at 38 °C for 24 h, the diameters of inhibition zone were measured using a metric ruler.

### β-Lactamase activity assay

The β-lactamase activity was evaluated using a nitrocefin hydrolysis assay [13]. The cultured cells were centrifuged at 3000×*g* for 20 min at 4 °C to collect cell-free supernatants. The cell-free supernatants were mixed with 20 μl of 1.5 mM nitrocefin (Biovision, Inc., CA, USA) and incubated at 37 °C. The absorbance was measured every 5 min up to 1 h at 515 nm [14].

### Ethidium bromide (EtBr)-agar cartwheel assay

The cultured *K*. *pneumoniae* cells were centrifuged at 3000×*g* for 20 min at 4 °C, rinsed twice with PBS, and then suspended in PBS with and without efflux pump inhibitor (phenylalanine-arginine-β-naphthylamide; PAβN, 8 μg/ml) [15, 16]. The prepared cells were swabbed on EtBr-agar plates containing EtBr (1 μg/ml) and incubated at 37 °C for 18 h. After incubation, the swabbed EtBr-agar plates exposed to UV light were photographed using a Gel-doc XR System (Bio-Rad, Hertfordshire, UK).

### Statistical analysis

All analyses were conducted in duplicates for three replicates. Data were analyzed by the Statistical Analysis System (SAS) software. The General Linear Model (GLM) and least significant difference (LSD) procedures were used to compare treatments at p < 0.05.

## Results

### Effect of serial antibiotic treatments on the viability and antibiotic susceptibility

**Fig. 1:**
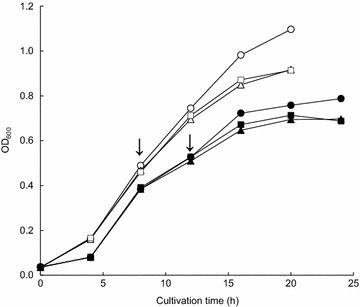
Survival of *Klebsiella pneumonia* exposed to sequential antibiotics (CON, *open circle*; CON-CIP, *open triangle*; CON-MER, *open square*; 1/4CIP-CON, *closed circle*; 1/4CIP-CIP, *closed triangle*; 1/4CIP-MER, *closed square*). *Arrow* indicates the addition time

**Fig. 2:**
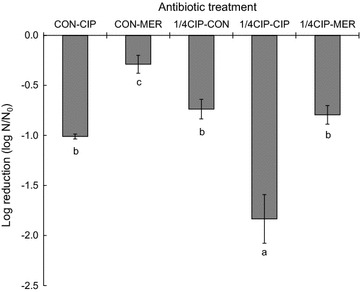
Reduction of *Klebsiella pneumonia* exposed to sequential antibiotics. *N*_CON_ and *N*_TRT_ represent the numbers of control and treatments after incubation, respectively. Log reduction with different letters (*a*–*c*) are significantly different at p < 0.05

**Fig. 3:**
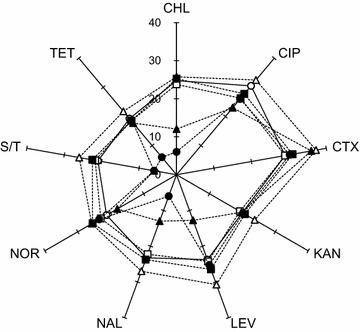
Radar plot of antibiotic resistance profiles (disk diffusion in cm) of *Klebsiella pneumonia* exposed to sequential antibiotics (CON, *open circle*; CON-CIP, *open triangle*; CON-MER, *open square*; 1/4CIP-CON, *closed circle*; 1/4CIP-CIP, *closed triangle*; 1/4CIP-MER, *closed square*). Antibiotic discs include chloramphenicol (CHL; 30 μg), ciprofloxacin (CIP; 5 μg), cefotaxime (CTX; 30 μg), kanamycin (KAN; 30 μg), levofloxacin (LEV; 5 μg), nalidixic acid (NAL; 30 μg), norfloxacin (NOR; 10 μg), sulphamethoxazole/trimethoprim (S/T; 25 μg), and tetracycline (TET; 30 μg)

The MIC values of ciprofloxacin and meropenem were 0.03 and 0.06 μg/ml, respectively. The survival of *K*. *pneumoniae* was observed during the sequential antibiotic treatments (Fig. 1). The growth of *K*. *pneumoniae* was retarded by 1/4 MIC ciprofloxacin, showing that the non- and ciprofloxacin-treated *K*. *pneumoniae* cells reached OD of 0.5 after 8 and 12 h of incubation, respectively. The antibiotic effects of ciprofloxacin and meropenem on the growth of *K*. *pneumoniae* was noticeable in the non-adapted cells (CON-CIP and CON-MER) compared to the ciprofloxacin-adapted cells (1/4CIP-CIP and 1/4CIP-MER). The number of viable *K*. *pneumoniae* cells under 1/4CIP-CIP was significantly reduced by 1.8 log, followed by CON-CIP and 1/4CIP-MER (Fig. 2). The least reduction in *K*. *pneumoniae* cells was observed at the CON-MER. The reduction rate increased with increasing the concentration of CIP to 0.03 μg/ml (1/4CIP-CIP), while no significant reduction was observed for the 1/4CIP-MER switched from CIP to MER at the second treatment. The antibiotic resistance acquisition was evaluated in *K*. *pneumoniae* exposed to sequential antibiotic treatments (Fig. 3). The susceptibilities of *K*. *pneumoniae* to different classes of antibiotics varied in the treatments. The 1/4CIP-CIP treatment exhibited the decreased susceptibility to all antibiotics with exception of CTX, showing small clear zones (<10 cm). The enhanced susceptibility to all antibiotics tested was observed at the CON-CIP treatment, while the decreased susceptibilities to CIP and TET were observed at the treatments, CON-MER, 1/4CIP-CON, and 1/4CIP-MER. The treatments, CON-MER and 1/4CIP-MER, showed the similar resistance pattern as the CON.

### Effect of sequential antibiotic treatments on β-lactamase and efflux pump activities

**Fig. 4:**
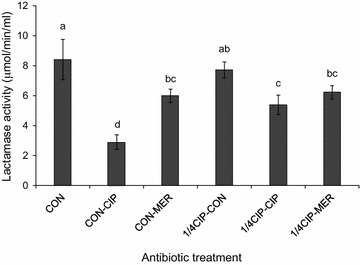
Hydrolyzing activity of the β-lactamases produced from *Klebsiella pneumonia* exposed to sequential antibiotics (CON, CON-CIP, CON-MER, 1/4CIP-CON, 1/4CIP-CIP, and 1/4CIP-MER). Means with different letters (*a*–*d*) are significantly different at p < 0.05

The β-lactamase activities were measured in *K*. *pneumoniae* cultured at sequential antibiotic treatments (Fig. 4). The highest β-lactamase activity was observed for the CON (8.4 μmol/min/ml), followed by 1/4CIP-CON (7.7 μmol/min/ml) and CON-MER (6.0 μmol/min/ml).

**Fig. 5:**
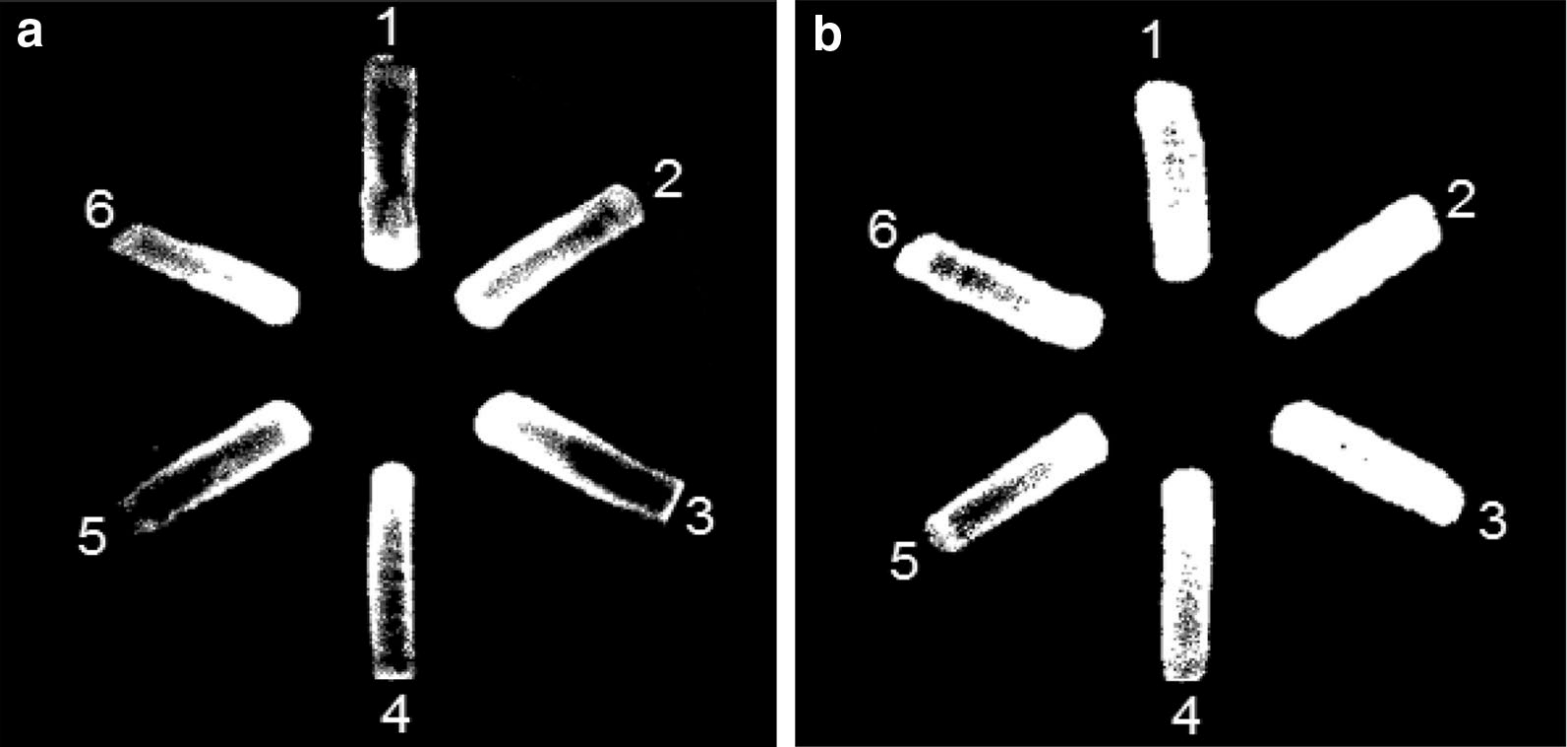
Accumulation and efflux activity of *Klebsiella pneumonia* on EtBr agar plates containing without (**a**) and with (**b**) efflux pump inhibitor (PAβN). (*1* CON; *2* CON-CIP; *3* CON-MER; *4*, 1/4CIP-CON; *5* 1/4CIP-CIP; *6* 1/4CIP-MER)

The efflux activity of *K*. *pneumoniae* in the absence and presence of efflux pump inhibitor (EPI; PAβN) was evaluated on the EtBr-agar plates (Fig. 5). The efflux pump activity of *K*. *pneumoniae* was observed at all treatments in the absence of PAβN, showing the decreased fluorescence intensity (Fig. 5a). The increased EtBr accumulation was observed in the presence of PAβN at the CON, CON-CIP, and CON-MER, while relatively low EtBr accumulation was observed at the 1/4CIP-CON, 1/4CIP-CIP, and 1/4CIP-MER (Fig. 5b). The efflux pump activity in *K*. *pneumoniae* cells treated at the 1/4CIP-CIP, 1/4CIP-CIP, and 1/4CIP-MER was not reduced by the PAβN, suggesting that the EPI-insensitive efflux systems were activated in *K*. *pneumoniae* cells under sublethal concentration of ciprofloxacin.

## Discussion

This study describes the effect of sequential antibiotic treatments on the development of antibiotic resistance in *K*. *pneumoniae*. Pathogenic bacteria exposed to different levels and various classes of antibiotics can exhibit various susceptibility to additional antibiotic treatments. However, few studies have been focused on the development of antibiotic resistance during the sequential antibiotic treatments [11]. Thus, this study highlights the pre-exposed antibiotic history as an important factor for the evolution of antibiotic resistance.

The CON-CIP was more effectively reduced the number of *K*. *pneumoniae* cells than the CON-MER, while no significant difference was observed between 1/4CIP-CON and 1/4CIP-MER (Fig. 2). The results indicate that meropenem was less effective against *K*. *pneumoniae* than ciprofloxacin. The lack of antibacterial activity of meropenem might be due to its short elimination half-life [17]. The successive ciprofloxacin treatment (1/4CIP-CIP) showed strong antibacterial activity against *K*. *pneumoniae* (Fig. 2). However, the cells exposed to 1/4CIP-CIP treatment induced were significantly resistant to different classes of antibiotics such as CHL, CIP, LEV, NAL, and S/T, known as cross-resistance induction (Fig. 3). This suggests that the sequential single-antibiotic therapy at different levels can lead to the emergence of antibiotic resistant bacteria due to the phenotypic adaptation [18]. Previous studies have reported that antibiotic monotherapy and combination therapy are the main causes of narrow- and broad-spectrum resistance, respectively [9]. The concentration of ciprofloxacin treated after the initial CIP exposure might be insufficient to suppress the development of antibiotic resistance [19]. The sequential antibiotic treatment, specifically 1/4CIP-CIP, can possibly cause a change in antibiotic resistance. This might be attributed to the mode of action of antibiotics, including time- and concentration-dependent activities. The time-dependent antibiotics include β-lactams and carbapenems, characterized by slow antibiotic activity according to exposure time above MIC [20, 21]. Ciprofloxacin is referred to as concentration-dependent antibiotic, showing antibiotic activity at high concentration [22]. Due to the bacterial adaptation, the sequential treatment of homogeneous antibiotic can reduce the susceptibility to same and different classes of antibiotics [19, 23]. Homogeneous treatment increases bacterial fitness and selection pressure, resulting in the development of antibiotic resistance [24, 25].

The strain of *K*. *pneumoniae* used in this study was intrinsically resistant to ampicillin (MIC > 256 μg/ml), piperazine (MIC > 16 μg/ml), cephalotin (MIC > 32 μg/ml), and cefoxitin (MIC > 16 μg/ml), resulted from the production of β-lactamases [26]. No significant change in β-lactamase activities was observed for 1/4CIP-CON when compared to the CON, while the β-lactamase activities were significantly decreased at the CON-CIP, CON-MER, 1/4CIP-CIP, and 1/4CIP-MER. The decrease in lactamase activity suggest that relatively fewer viable cells were observed at the treatments compared to the CON and the antibiotics used may act as a lactamase inhibitor [27]. *K*. *pneumoniae* cells exposed to CON-CIP showed the increased susceptibility to CTX (Fig. 3), which corresponds to the significant reduction in β-lactamase activity treated at CON-CIP (2.9 μmol/min/ml) (Fig. 4). The mechanisms of resistance to β-lactam antibiotics include not only the increase in β-lactamases-mediated hydrolysis but also the decrease in uptake through porin channels and the increase in efflux pump activity [6, 28]. The alteration in porin channel results in the decrease in membrane permeability to antibiotics such as β-lactams and quinolones, but not aminoglycosides that can enter the outer membrane through lipopolysaccharide (LPS) uptake system [19]. The results explain that the susceptibilities of *K*. *pneumoniae* cells grown at all treatments to KAN were constant regardless of the levels of β-lactamase activities (Fig. 3). Carbapenems such as imipenem and meropenem are stable to β-lactamases that can hydrolyze β-lactams [28–30]. The production of β-lactamases can contribute to the hydrolysis, resulting in the enhanced resistance to β-lactam antibiotics that cannot reach penicillin binding protein (PBP) [26]. This is in good agreement with the result of antibiotic susceptibility that *K*. *pneumoniae* possesses high intrinsic resistance to ampicillin (MIC > 256 μg/ml).

The efflux pump systems competitively expel substrates, which plays an important role in the development of multidrug resistance [2]. The specific substrates for efflux pumps in *K*. *pneumoniae* treated at 1/4CIP-CIP and 1/4CIP-CIP may include CHL, TET, NOR, NAL, and LEV, corresponding the enhanced multiple antibiotic resistance in disk-diffusion assay in Fig. 3. The intracellular concentrations of antibiotics substrates in bacteria are directly reduced by active efflux pump systems, leading to the decreased antibiotic susceptibility [31]. In other words, the inhibition of efflux pumps can restore antibiotic susceptibility in resistant bacteria, suggesting the EPI can be a possible control method against antibiotic-resistant bacteria [32–34]. The activation of efflux pumps is associated with the fast-acting antibiotic mechanism at the early stage of antibiotic resistance development [19]. The substrate-dependent efflux pump systems may contribute to cross-resistance to different classes of antibiotics [35].

## Conclusions

This study highlights the influence of using sequential antibiotic treatment on the development of antibiotic resistance in *K*. *pneumoniae*. The difference in antibiotic activity against *K*. *pneumoniae* might be due to the time- and concentration-dependent modes of action of meropenem and ciprofloxacin, respectively. The antibiotic resistance mechanisms can occur within a short time in association with lactamase production, membrane permeability, and efflux pump activity. The efflux pump activities in *K*. *pneumoniae* cells treated at 1/4CIP-CON, 1/4CIP-CIP, and 1/4CIP-MER were still observed in the presence of PAβN, indicating that various types of substrate-dependent efflux pumps exist to reduce the intracellular concentrations of antibiotics. The induced efflux pump activity are responsible for the increased antibiotic resistance in *K*. *pneumoniae*. The most significant finding in this study was that *K*. *pneumoniae* treated with sequential antibiotics, specifically 1/4CIP-CIP, showed reduced susceptibility towards CHL, NOR, NAL, and LEV, leading to the multiple antibiotic resistance. Therefore, antibiotic therapies should take into account the history of pre-exposed antibiotics to prevent the development of antibiotic resistance. Further studies are needed to assess the risk of antibiotic resistance in sequential and combination antibiotic therapies, which is essential to design an effective strategy for controlling multiple antibiotic resistant bacteria.

## Abbreviations

ESBL: extended-spectrum β-lactamase
MIC: minimum inhibitory concentration
CIP: ciprofloxacin
MER: meropenem
CTX: cefotaxime
CHL: chloramphenicol
KAN: kanamycin
LEV: levofloxacin
NAL: nalidixic acid
NOR: norfloxacin
S/T: sulphamethoxazole/trimethoprim
TET: tetracycline
CFU: colony forming unit
EtBr: ethidium bromide
EPI: efflux pump inhibitor
PAβN: phenylalanine-arginine-β-naphthylamide
